# HOXA5 confers tamoxifen resistance via the PI3K/AKT signaling pathway in ER-positive breast cancer

**DOI:** 10.7150/jca.59740

**Published:** 2021-06-01

**Authors:** Clara Yuri Kim, Yu Cheon Kim, Ji Hoon Oh, Myoung Hee Kim

**Affiliations:** 1 Department of Anatomy, Embryology Laboratory, Yonsei University College of Medicine, Seoul 03722, Korea.; 2 Department of Anatomy, Graduate School of Medical Science, Bain Korea 21 Project, Yonsei University College of Medicine, Seoul 03722, Korea.

**Keywords:** HOXA5, AKT, tamoxifen resistance, breast cancer, p53

## Abstract

Tamoxifen is a commonly used drug to treat estrogen receptor-positive patients with breast cancer. Despite the outstanding efficacy of tamoxifen, approximately one-third of patients develop resistance toward it, thereby presenting a therapeutic challenge. *HOX* genes may be involved in the acquisition of tamoxifen resistance. In this study, we identified HOXA5, a member of the HOX gene family, as a marker of tamoxifen resistance. Using ChIP assay, we found that HOXA5 expression was significantly overexpressed in tamoxifen-resistant MCF7 (TAMR) breast cancer cells because of reduced H3K27me3 binding. HOXA5 upregulation resulted in activation of the PI3K/AKT signaling cascade, which in turn, led to p53 and p21 reduction, ultimately making the TAMR cells less apoptotic. Furthermore, elevated HOXA5 expression resulted in breast cancer cells acquiring more mesenchymal-like and stem cell traits associated with aggressive breast cancer phenotypes. In conclusion, our results delineate a mechanism by which HOXA5 promotes tumorigenesis, cancer progression, and tamoxifen resistance in breast cancer cells.

## Introduction

Cancer is characterized by uncontrolled cell division that commonly turns malignant and metastasizes by migrating and invading other healthy tissues in the body 1, 2. Among various types of cancer, breast cancer is one of the most prevalent cancers worldwide 3-5. It involves the formation of malignant tumors in the breast tissues, usually in the ducts of the terminal duct lobular unit 6. Breast cancer can be classified into four different molecular subtypes: luminal A (estrogen receptor/progesterone receptor-positive [ER/PR+], human epidermal growth factor receptor 2-negative [HER2-]), luminal B (ER/PR+, HER2+), triple negative (ER/PR-, HER2-), and HER2-enriched (ER/PR-, HER2+) 7, of which the ER+ subtype is the most prevalent. ER+ cases are typically treated with drugs belonging to the class of selective ER modulators (SERMs) 8, 9. Tamoxifen, one of the most commonly administered SERMs, competes with estradiol, a major estrogen sex hormone for ER binding, to function as a partial ER antagonist and a transcriptional inhibitor of estrogen-responsive genes, thereby inhibiting breast cancer growth. The robustness of tamoxifen makes it an excellent drug for administration in both pre- and post-menopausal females, as well as in male patients with breast cancer 10. However, approximately one-third of patients eventually develop resistance to tamoxifen, ultimately leading to relapse and eventually metastatic breast cancer 11-14. Although there are extensive studies on the mechanisms underlying the development of tamoxifen resistance, knowledge on molecular markers that can predict tamoxifen resistance and thus breast cancer relapse is still lacking. Therefore, it is imperative to identify novel biomarkers of tamoxifen resistance to overcome major obstacles in improving existing therapies for the treatment and prevention of breast cancer.

The homeobox (*HOX*) genes are a group of highly conserved genes organized into four different clusters: *HOXA*,* HOXB*,* HOXC*, and *HOXD*. These genes play central roles in encoding transcription factors involved in developing the anterior-posterior axis during embryogenesis 15-20. The collinear expression of *HOX* genes has been extensively studied to reveal the mechanism underlying sequential patterning of the body axis and secondary axial structures of an embryo in space and time 20-22. More recently, dysregulated *HOX* expression has been identified in cancer 23-26. The altered expression of *HOX* genes has been studied in various cancers such as colon, lung, ovarian, prostate, and breast cancer 27-32, wherein it may play an oncogenic or tumor-suppressive role. Interestingly, specific *HOX* genes may play both oncogenic and tumor-suppressive roles in a tumor site-specific manner. Abnormal expression of certain *HOX* genes can promote tumorigenesis by evading apoptosis, altering signaling pathways, and promoting epithelial-mesenchymal transition (EMT). In contrast, other *HOX* genes can induce differentiation of cancer cells to prevent tumor growth and proliferation 33, 34. Nevertheless, the precise functional mechanism of *HOX* genes in regulating tamoxifen resistance in breast cancer is yet to be elucidated.

Many *HOX* dysregulations have been explained through epigenetic mechanisms 16, 35. The expression of *HOX* genes is commonly controlled by epigenetic regulations such as DNA methylation and histone modifications during normal development or cancer 16, 35-37. In particular, histone modifications regulated by histone methyltransferases, histone demethylases, and histone acetyltransferases can result in dynamic conformational changes in the chromosome, which results in discrepant *HOX* expression 16, 35-38.

In this study, we aimed to investigate the molecular mechanisms underlying the role of HOXA5 in tamoxifen resistance. Using a systematic *in vitro* approach to analyze the function of HOXA5, we found that higher levels of HOXA5 in tamoxifen-resistant MCF7 (TAMR) breast cancer cells activated the PI3K/AKT signaling pathway and resulted in reduced p53 and p21 levels and significantly reduced proportion of apoptotic cells after tamoxifen treatment. Enhanced metastasizing capabilities and an enrichment of breast cancer stem cells were also associated with HOXA5 overexpression. Overall, our results revealed the mechanism by which HOXA5 acts as an important factor in the acquisition of tamoxifen resistance and enhancement of breast cancer aggressiveness and implied that modulation of HOXA5 expression and its related downstream pathways can demonstrate noteworthy benefits for patients experiencing drug resistance and disease recurrence.

## Materials and methods

### Cell lines and culture

MCF7 and MCF7-TAMR cells were cultured in Dulbecco's modified Eagle's medium (WelGENE Inc., Daegu, Korea). The medium was supplemented with heat-inactivated 10% FBS (WelGENE Inc.) and 1% penicillin-streptomycin (WelGENE Inc.). In case of the MCF7-TAMR cells, the medium additionally contained 1 μM 4-hydroxytamoxifen (Sigma, MO, USA). All cells were grown at 37 °C in a 5% CO_2_ incubator. TAMR cells were generated as *in vitro* models of acquired tamoxifen resistance by exposing the parent cell lines MCF7 for a long-term with 1 μM 4-hydroxytamoxifen (Sigma) treatment. For overexpression studies, MCF7 cells were transfected with the HOXA5 plasmid (pCMV6-AC-GFP vector backbone; OriGene Technologies, Inc., Rockville, MD, USA) for 24 h using the Attractene transfection reagent (Qiagen, Hilden, Germany) according to the manufacturer's protocol. Knockdown studies were performed by transfecting TAMR cells for 24-48 h with 40 nM siHOXA5 using G-fectin (Genolution, Seoul, Korea) following the manufacturer's protocol. A pool of 5 individual siRNAs targeting exons 1 and 2 of *HOXA5* were used for the knockdown.

### Reverse transcription (RT)-qPCR

Total RNA was extracted from cells using TRIzol reagent (Invitrogen, CA, USA), and cDNA was synthesized with 1 μg of total RNA using ImProm-II^TM^ Reverse Transcriptase (Promega, WI, USA). Reverse transcription was performed under the following conditions: initial denaturation for 5 min at 94 °C, followed by 27-35 cycles of 94 °C for 40 s (depending on target genes), 58 °C for 20 s, and 72 °C for 30 s. For quantitative PCR, the StepOnePlus^TM^ Real-Time PCR System (Applied Biosystems, CA, USA) and Power SYBR Green PR Master Mix (Applied Biosystems) were used. All qPCR reactions were performed in at least three independent biological replicates, and β-Actin and GAPDH were used as internal controls. The RT-PCR primers are listed in **Table 1**.

### Western blotting

MCF7 and TAMR cells were treated under the appropriate conditions and then lysed with NP-40 (Biosesang, Sungnam, Korea), after which their protein concentrations were determined using the Pierce BCA Protein Assay Kit (Thermo Scientific, MA, USA). Each protein sample was loaded onto 8-10% SDS polyacrylamide gel, and then electro transferred to a PVDF transfer membrane (BioRad, CA, USA). Immunoreactive bands were detected using target primary antibodies and corresponding HRP-conjugated secondary antibodies. Bands were visualized using the SuperSignal West Pico Chemiluminescent Substrate Kit (Thermo Scientific). Anti-HOXA5 (ab82645; Abcam), anti-EZH2 (#5246S; Cell Signaling, MA, USA), anti-SUZ12 (#3737; Cell Signaling), anti-EED (ab96801; Abcam, Cambridge, UK), UTX (ab36938; Abcam), anti-JMJD3 (ab169197; Abcam), anti-AKT (#2967; Cell Signaling), anti-phospho-AKT (Thr308, #9275; Cell Signaling), anti-phospho-AKT (Ser473, #4508; Cell Signaling), anti-p53 (sc-126; Santa Cruz Biotechnology, CA, USA), anti-p21 Waf1/Cip1 (#2947; Cell Signaling), anti-Caspase-7 (#12827; Cell Signaling), anti-cleaved-Caspase-7 (#8438; Cell Signaling), anti-Caspase-9 (#9508; Cell Signaling), anti-cleaved-Caspase-9 (#52873; Cell Signaling), anti-PARP (#9542; Cell Signaling), anti-cleaved-PARP (#5625; Cell Signaling), anti-E-cadherin (ab40772; Abcam), anti-N-cadherin (ab18203; Abcam), anti-ZEB1(ab181451; Abcam), and anti-β-Actin (ab6276; Abcam) antibodies were used to detect each of the respective proteins.

### Cell proliferation assay

Relative cell proliferation was measured using the Cell Counting Kit-8 (Dojindo Molecular Technologies Inc., Kumamoto, Japan) following the manufacturer's protocol. Briefly, 7.5 × 10^3^ cells/well were plated and grown on 96-well plates, stained with 10 μL of WST-8, and incubated for 3 h at 37 °C in a 5% CO_2_ incubator for three consecutive days. The plate was then measured for absorbance at 450 nm using a Softmax Pro microplate reader (Molecular Devices, CA, USA).

### Apoptosis analysis

The EzWay Annexin V-FITC Apoptosis Detection Kit (Komabiotech, Seoul, Korea) was used to detect apoptosis. Breast cancer cells (3 × 10^5^ cells per cell line) were harvested, centrifuged, and washed twice with cold Phosphate Buffered Saline (PBS). The washed cells were resuspended in 500 μL of cold binding buffer. Subsequently, 1.25 μL (200 μg/mL) of the Annexin V-FITC reagent was added and incubated at room temperature in the dark for 15 min. Cells were washed with 500 μL of cold binding buffer, then 10 μL of PI (30 μg/mL) was added, and finally analyzed by flow cytometry.

### Chromatin immunoprecipitation (ChIP) analysis

For ChIP analysis, cells were fixed with 1% formaldehyde for 15 min at room temperature, and then quenched with 2.5 M glycine. Subsequently, the cells were lysed on ice for 10 min in SDS buffer containing protease inhibitors, and then sonicated with Sonics Vibra Cell^TM^ (Sonics & Materials Inc., CT, USA; 7 min: 10 s pulse, 10 s interval) on ice. The fragmented chromatin samples were centrifuged at 8000 ×*g* at 4 °C for 5 min and the supernatant was collected. The samples were pre-cleared, and then incubated overnight at 4 °C with the appropriate antibodies and protein-coated A/G agarose beads (Santa Cruz) with gentle shaking. The immunoprecipitated eluates were reverse cross-linked and recovered through DNA purification for PCR. Anti-H3K4me3 (ab1012; Abcam), anti-H3K9ac (ab12179; Abcam), anti-H3K27me3 (ab6002; Abcam), anti-EZH2 (#5246S; Cell Signaling), anti-SUZ12 (#3737; Cell Signaling), anti-EED (ab96801; Abcam), anti-UTX (ab36938; Abcam), anti-JMJD3 (ab169197; Abcam), and non-immune mouse IgG (sc2025; Santa Cruz) antibodies were used. ChIP-PCR primers are listed in **Table 2**.

### Invasion and migration analyses

For the invasion assay, cells were harvested and resuspended in serum-free media, and 100 μL of cell suspension (5 × 10^4^ cells) were seeded into inserts that were pre-coated with Matrigel (BD, CA, USA) (150 mg/mL) mixed with coating buffer. Standard media with 10% FBS was added to the bottom of the wells. After 72 h, the cells on the upper surface of the inserts were removed with cotton swabs, and the cells that invaded the bottom of the inserts were fixed with methanol and stained with DAPI. For the migration assay, the same protocol was used in the absence of Matrigel. Cells were observed and imaged by fluorescent microscopy, and cells were counted using Image J software.

### Spheroid formation assay

Spheroid cultures were grown as described, with minor changes 39. Briefly, 1 × 10^4^ cells/mL were counted and resuspended carefully using a 25G syringe needle to obtain a single-cell suspension. The cells were pelleted, washed with cold PBS, and syringe-filtered again to ensure that it remains as a single-cell suspension. Cells were then plated onto Ultra-Low attachment 6-well plates (Corning, NY, USA) with 2 mL DMEM/F12 media (WelGENE Inc.) supplemented with 1% PSA, 2% B27, 10 ng/mL FGFb, 20 ng/mL EGF, 5 μg/mL insulin, and 4 μg/mL heparin. Cells were maintained at 37 °C in a 5% CO_2_ incubator. The number of spheroids with a diameter greater than 50 μm was regularly counted, and when there were approximately 100 spheroids of this diameter, they were collected by gentle centrifugation, dissociated, and then passaged for the assessment of self-renewal.

### In silico analysis

The web-accessible database cBioPortal (http://www.cbioportal.org) was used to assess *HOXA5* abnormalities in breast cancer tissues. The Gene Expression across Normal Tumor Tissue (GENT) publicly available database (http://medical-genome.kribb.re.kr/GENT) was used to evaluate *HOXA5* gene expression patterns in breast cancer tissues. The Kaplan-Meier plotter (http://www.kmplot.com) was used for survival analysis. This database allows for the assessment of 54,675 genes on overall survival (OS) and distant metastasis-free survival (DMFS). To investigate the prognostic values of the* HOXA5* gene, patient samples were classified into low- and high-expression groups, using the median as the auto select best cutoff.

### Statistical analysis

All data are expressed as mean ± SD. Statistical differences were determined using Student's *t*-test or one-way ANOVA for pairwise comparisons. A *p*-value of < 0.05 was considered as statistically significant.

## Results

### Elevated expression of HOXA5 is associated with tamoxifen resistance in breast cancer

**Figure 1:**
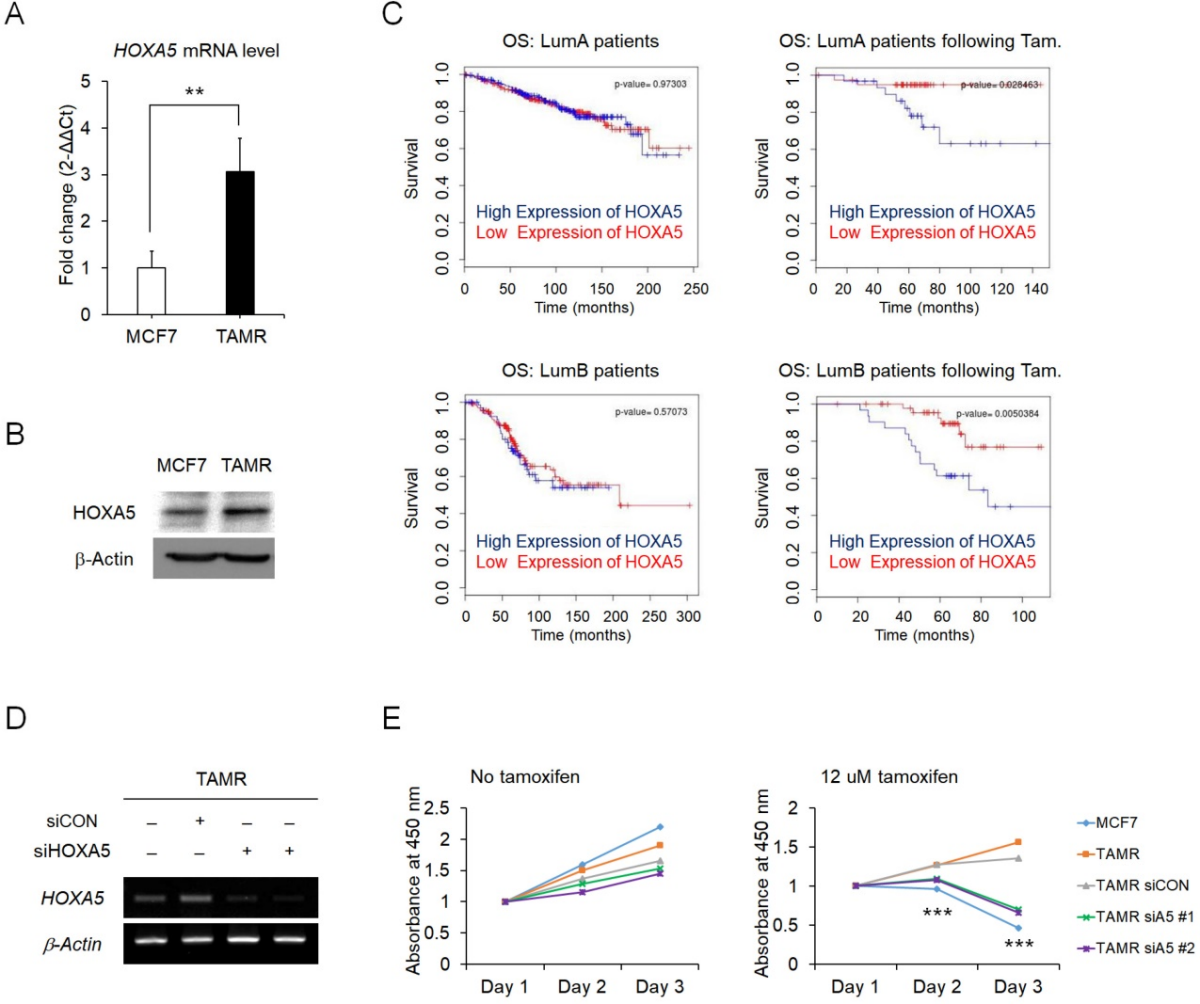
** Elevated expression of HOXA5 is associated with tamoxifen resistance in MCF7 breast cancer cells.** (A) Reverse transcription-quantitative PCR (RT-qPCR) analysis of *HOXA5* expression in MCF7 and tamoxifen-resistant breast cancer (TAMR) cells. GAPDH was used to normalize changes in *HOXA5* expression levels between the two cell lines. (B) Western blotting of HOXA5 in MCF7 and TAMR cells. (C) Kaplan-Meier analysis of overall survival (OS) in estrogen receptor-positive (Luminal A and Luminal B) breast cancer patients with and without tamoxifen treatment. (D) RT-PCR analysis of *HOXA5* in TAMR cells transiently transfected with pooled siHOXA5 for 48 hrs. (E) Cell viability curve of cells treated with siHOXA5 in the absence (left panel) and presence of 12 μM tamoxifen (right panel) at days 1, 2, and 3. β-Actin was used as an internal control for RT-PCR and western blotting. All experiments were performed in triplicate. ** *p* < 0.01, *** *p* < 0.001 compared with siCON by Student's *t*-test.

To identify the regulatory molecules involved in acquired tamoxifen resistance, we compared the expression levels of the complete set of *HOX* genes in between MCF7 and TAMR cells via RT-qPCR. Amongst the genes showing differential expression levels between the two cell strains, *HOXA5* was one of the genes which showed a dramatic increase in the expression levels in the tamoxifen-resistant breast cancer cells (**Fig. 1A**). Correspondingly, the protein levels of HOXA5 also showed a consistent upregulation in TAMR cells (**Fig. 1B**). More importantly, survival curves from ER+ breast cancer patients without tamoxifen treatment were independent of *HOXA5* expression as compared to survival curves of patients who received tamoxifen treatment, which demonstrated that a higher expression of *HOXA5* resulted in a poorer overall survival (**Fig. 1C**). To determine whether tamoxifen resistance acquired by the cells is a direct consequence of *HOXA5* abundance, *HOXA5* was knocked down in TAMR cells by multiple small interfering RNAs (siRNAs) (**Fig. 1D**). The functional significance of *HOXA5* knockdown was confirmed by the CCK-8 cell viability assay, which showed that the loss of *HOXA5* expression was sufficient to re-sensitize TAMR cells to a high-dose of tamoxifen treatment (**Fig. 1E**).

### Epigenetic regulation of* HOXA5* occurs at its putative promoter region

**Figure 2:**
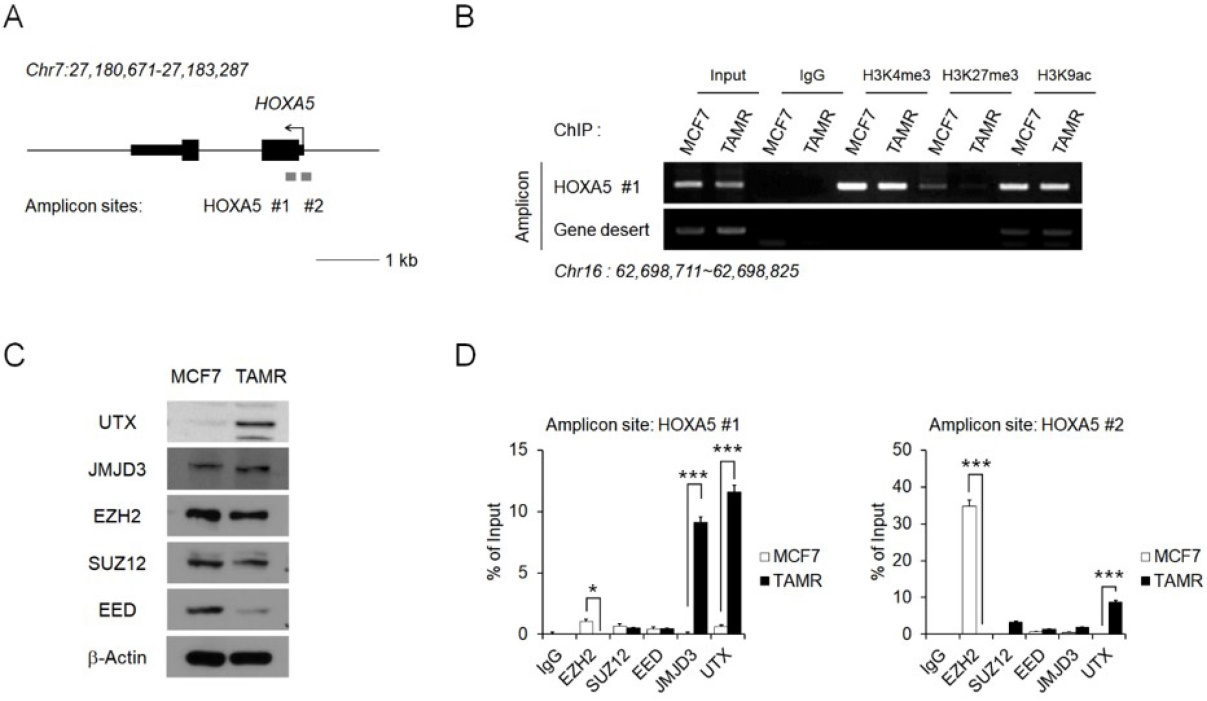
** Epigenetic regulation of* HOXA5* occurs at its putative promoter region.** (A) Schematic depiction of the *HOXA5* locus on human chromosome 7. Boxes represent exons, lines represent introns, and arrows show the direction of transcription. The gray bars represent the amplicon sites used in chromatin immunoprecipitation-PCR (ChIP-PCR). (B) ChIP-PCR analysis of the histone modifications H3K4me3, H3K27me3, and H3K9ac in MCF7 and TAMR cells. (C) Western blotting images showing protein levels of the epigenetic modifiers in MCF7 and TAMR cells. β-Actin was used as an internal control. (D) ChIP-PCR analysis of EZH2, SUZ12, EED, JMJD3, and UTX in MCF7 and TAMR cells. All experiments were performed in triplicate. *** *p* < 0.001 compared with MCF7 by Student's *t*-test.

To investigate the reason behind *HOXA5* upregulation in TAMR cells, epigenetic modifications present at the *HOXA5* putative promoter region were examined via chromatin immunoprecipitation (ChIP) coupled with quantitative PCR (ChIP-PCR) assay (**Fig. 2A**). In particular, histone modifications and their binding affinities to the putative *HOXA5* promoter region were studied. Active histone marks such as histone H3 lysine 4 trimethylation (H3K4me3) and histone H3 lysine 9 acetylation (H3K9ac) were enriched at similar levels between MCF7 and TAMR cells. Interestingly, histone H3 lysine 27 trimethylation (H3K27me3), a well-known repressive marker, could not be found in TAMR cells (**Fig. 2B**).

We therefore analyzed factors involved in the binding dynamics of H3K27me3 in cells. Levels of the PRC2 complex components (EZH2, SUZ12, and EED) and the histone demethylases (JMJD3 and UTX), along with their protein levels were examined in MCF7 and TAMR cells. While the protein levels of EZH2, SUZ12, and JMJD3 were comparable between the two cell lines, the protein level of EED—the core component of the PRC2 complex, was downregulated in TAMR cells, whereas the level of UTX—a major histone demethylase, was upregulated in TAMR cells (**Fig. 2C**). To further test whether these histone modifiers are involved in the epigenetic regulation of *HOXA5* expression, their enrichment at the *HOXA5* promoter region was confirmed by ChIP-PCR (**Fig. 2D**). At the first amplicon site, the binding of JMJD3 and UTX was dramatically higher in TAMR cells whereas, the binding of EZH2 was much higher at the second amplicon site in MCF7 cells (**Fig. 2D**).

To validate whether the transcriptional activation of *HOXA5* is directly regulated by the binding of JMJD3 and UTX at its promoter, we examined the effect of GSK-J4, a JMJD3/UTX inhibitor, on *HOXA5* expression in MCF7 and TAMR cells. Upon GSK-J4 treatment, the expression level of *HOXA5* was unchanged in MCF7 cells compared to DMSO-treated control, since JMJD3 is not bound at the *HOXA5* promoter in MCF7 cells. On the other hand, a significant reduction in *HOXA5* expression was observed in TAMR cells when JMJD3 and UTX histone demethylase binding was inhibited using GSK-J4. The expressions of NANOG and OCT4 were confirmed as positive and negative controls as NANOG showed reduced expression, but OCT4 remained unchanged with GSK-J4 treatment in a previous study 40 (**Fig. S1**). Altogether, these results suggest that the transcription of *HOXA5* receives epigenetic regulation from multiple factors, and is directly regulated by the binding of JMJD3 and UTX at its promoter region, thereby explaining its differential expression observed between MCF7 and TAMR cells.

### HOXA5 downregulates p53/p21 expression via activation of the PI3K/AKT signaling pathway in TAMR cells

**Figure 3:**
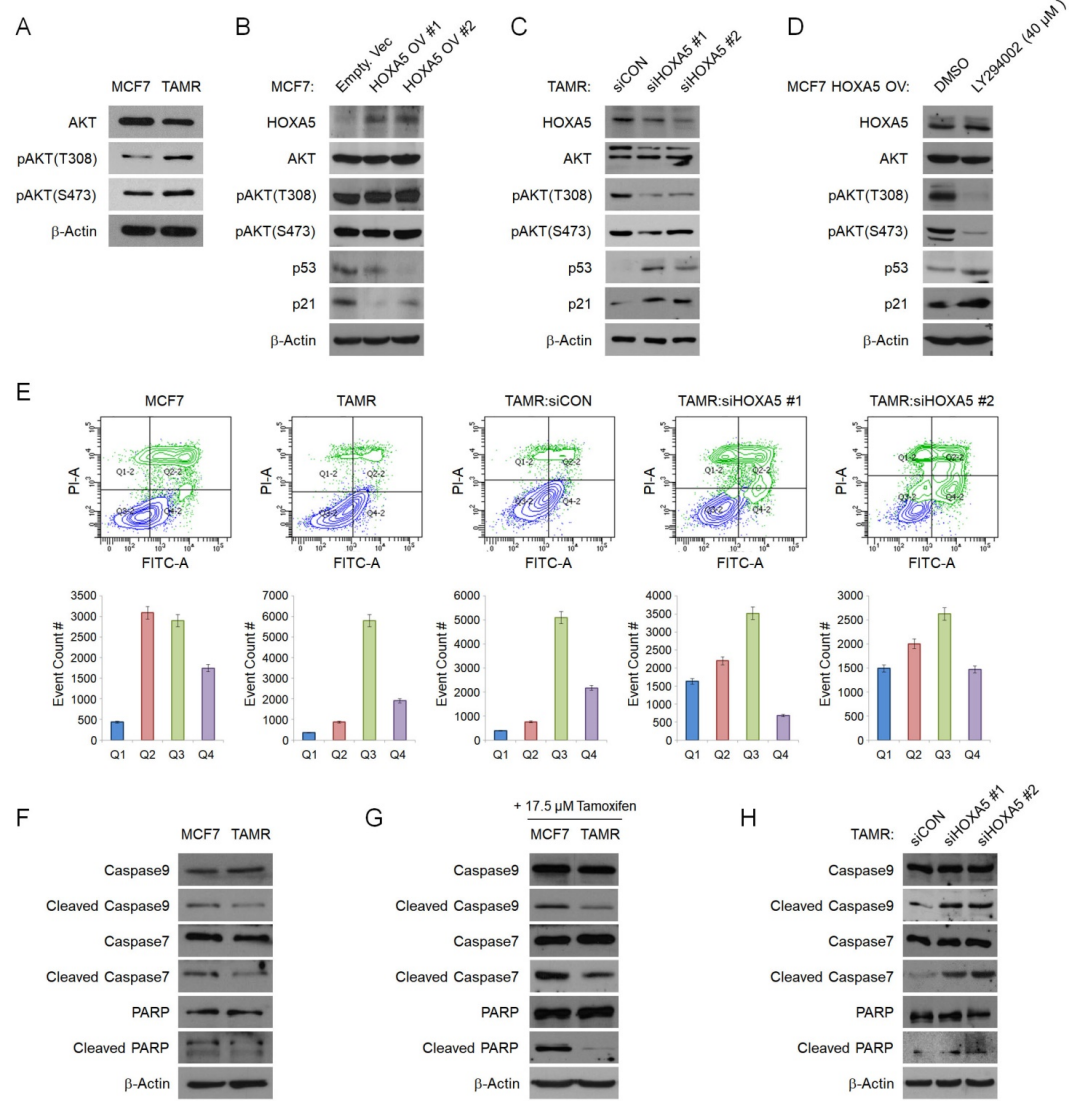
** HOXA5 downregulates p53/p21 expression via activation of the PI3K/AKT signaling pathway in TAMR cells.** (A) Western blotting for basal PI3K/AKT activity between parent MCF7 and TAMR cells. (B) Western blotting for PI3K/AKT pathway activation and the downstream p53 and p21 expression in MCF7 cells transfected with either the empty vector or HOXA5-overexpressing plasmids. (C) Western blotting for PI3K/AKT activation and downstream p53 and p21 expression in TAMR cells transfected with control or HOXA5 siRNAs. (D) Western blotting of PI3K/AKT activity and p53/p21 levels in HOXA5-overexpressing MCF7 cells after treatment with DMSO and LY294002 - a highly selective PI3K inhibitor for 24 hrs. (E) Representative scatter plots and quantification graphs showing the distribution of Annexin V and Propidium iodide (PI) staining from flow cytometry analysis. Parent cell lines and TAMR cells transfected with control or HOXA5 siRNAs were used for this analysis. Cells are classified as “viable” (Q3; bottom left), “early apoptotic” (Q4; bottom right), “late apoptotic” (Q2; top right), or “necrotic” (Q1; top left). (F) Western blotting for basal protein levels of full-length and cleaved caspases and PARP in parent MCF7 and TAMR cells. (G) Western blotting for the cleaved forms caspases and PARP after tamoxifen treatment in parent MCF7 and TAMR cells. (H) Western blotting for the cleaved forms of caspases and PARP after HOXA5 knockdown in TAMR cells. β-Actin was used as an internal control. All experiments were performed in triplicate.

To elucidate the mechanism of HOXA5 in tamoxifen resistance, we sought to identify the signaling pathways involving HOXA5. We utilized the cBioPortal Pathway Mapper to list altered signaling pathways associated with altered levels of *HOXA5* in breast cancer. Among the listed pathways, the PI3K/AKT and TP53 pathways were ranked top-most (**Fig. S2A** and **S2B**). Other signaling pathways such as WNT, NOTCH, MYC, and TGFβ showed less correlation with *HOXA5* alteration frequencies (data not shown). Consequently, we checked the protein levels of AKT and phosphorylated AKT (pAKT) in MCF7 and TAMR cells by western blotting. TAMR cells showed enhanced basal levels of both pAKT^T308^ and pAKT^S473^ when compared to MCF7 cells (**Fig. 3A**). Based on the above observations, we hypothesized that HOXA5 plays a key role in activating the AKT signaling cascade. To test this hypothesis, HOXA5 was overexpressed in MCF7 cells and silenced in TAMR cells. Remarkably, HOXA5 overexpression led to increased steady-state levels of AKT activity in MCF7 cells, which had initially showed basal AKT activity in the naïve state. Further, activation of the AKT signaling cascade in HOXA5-overexpressing MCF7 cells resulted in reduced protein levels of p53 and p21, the downstream effectors of the AKT pathway (**Fig. 3B**). Moreover, the inhibition of HOXA5 in TAMR cells was sufficient to override the hyperactive AKT signaling, especially at the T308 locus. Furthermore, de-activation of the AKT signaling pathway restored the expression of p53 and p21 (**Fig. 3C**).

To additionally re-confirm that the AKT signaling pathway is activated in response to the elevated levels of HOXA5 and this activation is key to the reduced levels of p53/p21, HOXA5-overexpressing MCF7 cells were treated with a highly selective PI3K inhibitor - LY294002, which has already been shown to block PI3K-dependent AKT phosphorylation. Upon treatment with the inhibitor, AKT activity induced by HOXA5 overexpression was successfully inhibited, resulting in the p53 and p21 expression levels to be rescued (**Fig. 3D**). Collectively, these data support our hypothesis of the functional role of HOXA5 in tamoxifen resistance by regulation of the PI3K/AKT pathway. Our data also bolsters the point that AKT signaling is an important regulator of tumor survival in the presence of tamoxifen in breast cancer.

Since p53 and p21 levels were affected by HOXA5 expression, we analyzed the role of HOXA5 in apoptosis. Flow cytometry analyses were used to compare the levels of apoptosis between parent MCF7 and TAMR cells in the absence and presence of high-dose tamoxifen treatment. Approximately 50% of MCF7 cells underwent apoptosis when exposed to high-dose tamoxifen as compared to TAMR cells which showed insignificant changes. Further analyses revealed that knockdown of HOXA5 in TAMR cells also dramatically increased the apoptotic population upon tamoxifen treatment compared to that of control cells (**Fig. 3E**). To determine whether the increase in the apoptotic activity of the cells is a direct consequence of the elevated HOXA5 levels, the expressions of pro-apoptotic proteins were explored. Elevated levels of caspases, PARP, and their cleaved products are considered hallmarks of apoptosis. Cleavage activates caspases which then act by cleaving a variety of substrates, including PARP and ultimately leading to cell death. Hence, protein levels of caspase 9, caspase 7, PARP, and their respective cleaved forms were investigated. Caspase 3 was excluded because it is not expressed in MCF7 cells. The basal levels of the inactive full-length caspases and PARP were found to be similar between MCF7 and TAMR cells; however, levels of cleaved caspases and PARP were much higher in MCF7 cells compared to TAMR cells. This pattern in the expression levels stayed consistent even under untreated conditions (**Fig. 3F**). The differential expression between the full-length and the cleaved forms became even more apparent upon high-dose tamoxifen treatment which showed that the levels of cleaved caspases and PARP were upregulated in tamoxifen-treated MCF7 cells, explaining their sensitivity to tamoxifen, hence increasing the apoptotic cell population (**Fig. 3G**). In addition, when HOXA5 was depleted from TAMR cells, the molecular levels of cleaved caspases and PARP were increased to similar levels seen in the MCF7 cells, indicative of impaired DNA repair function (**Fig. 3H**). Combined, these results demonstrate that elevated HOXA5 expression activates the AKT signaling pathway, which consequently reduces p53 and p21 levels, ultimately leading to a less apoptotic-prone and tamoxifen-resistant phenotype of the cell.

### HOXA5 mediates metastatic abilities and stemness of breast cancer cells resulting in aggressive phenotypes

**Figure 4:**
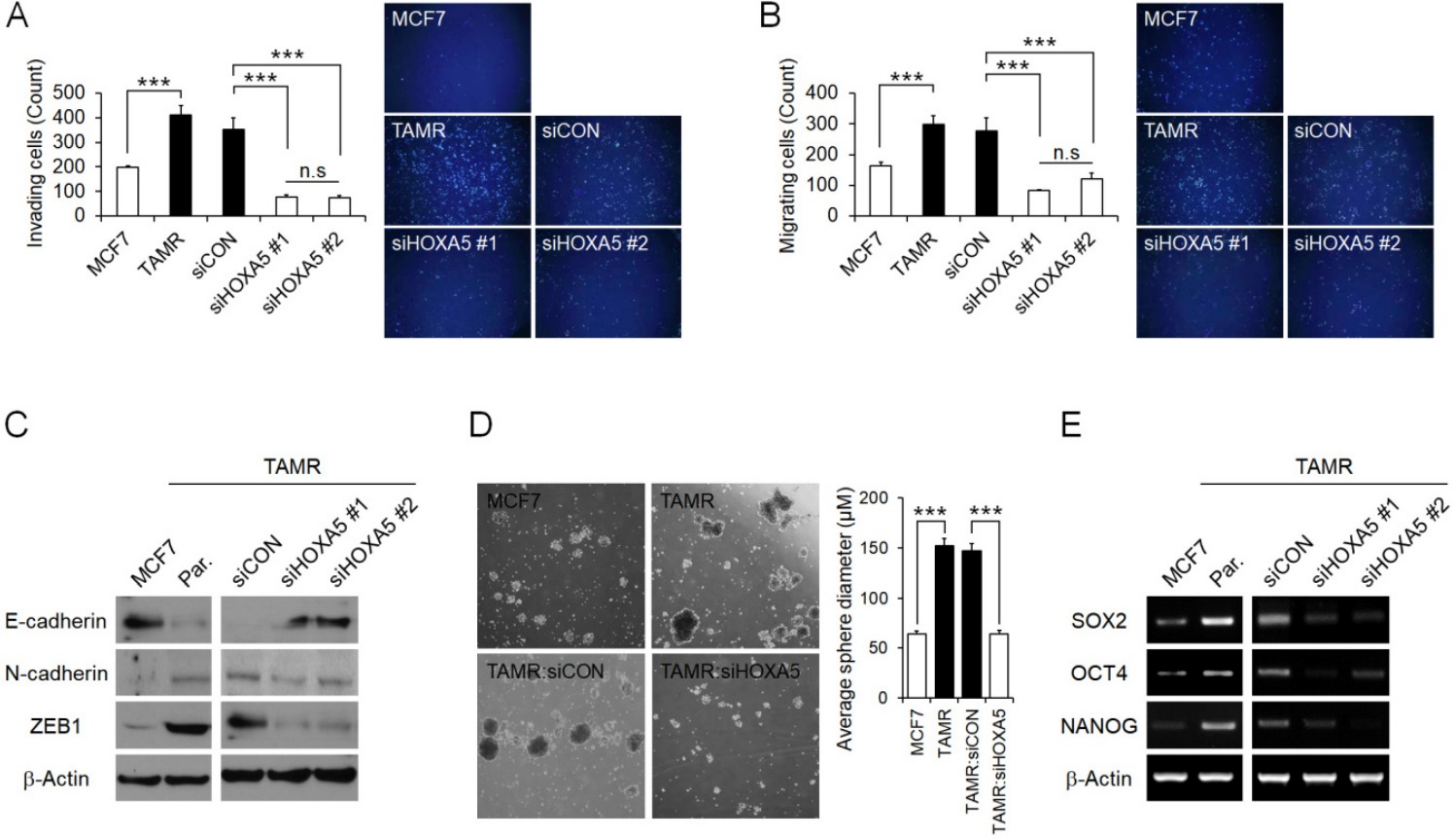
** HOXA5 mediates invasion and migration abilities and stemness of breast cancer cells and induces aggressive phenotypes.** (A) Matrigel invasion assay in parent MCF7, TAMR, and TAMR cells transfected with control or HOXA5 siRNAs. Representative images acquired by fluorescent microscopy after DAPI staining are shown. Quantitative interpretation was attained using ImageJ. (B) Migration assay in parent MCF7, TAMR, and TAMR cells transfected with control or HOXA5 siRNAs. Representative images acquired by fluorescent microscopy after DAPI staining are shown. Quantitative interpretation was attained using ImageJ. (C) Western blotting for epithelial and mesenchymal markers in parent MCF7, TAMR, and TAMR cells transfected with control or HOXA5 siRNAs. (D) Left panel shows representative microscopic images of breast cancer stem cell (BCSC) sphere growth of parent MCF, TAMR, and TAMR cells transfected with control or HOXA5 siRNAs. Right panel shows graphic presentation of the average sphere diameter. The data were retrieved and analyzed on the 14^th^ day of spheroid culture. (E) Reverse transcription-quantitative PCR analysis of *SOX2*, *OCT4*, and *NANOG* in parent MCF7, TAMR, and TAMR cells transfected with control or *HOXA5* siRNAs. All experiments were performed in triplicate. *** *p* < 0.001 compared with MCF7 and siCON respectively by Student's t-test or one-way ANOVA.

Next, the functional role of HOXA5 resulting in aggressive phenotypes in breast cancer cells associated with tamoxifen resistance was assessed. MCF7 cells have been reported to display poor invasiveness, whereas TAMR cells have enhanced invasive and migratory characteristics 41. Therefore, we initially examined the invasion and migration abilities of the parent MCF7 and TAMR cells, as well as TAMR cells that have undergone siRNA-mediated HOXA5 knockdown. As expected, parent TAMR cells had considerably higher invading and migrating cell populations than parent MCF7 cells (**Fig. 4A** and **4B**). Consequently, HOXA5-depleted TAMR cells displayed a dramatic diminishment in the invasive and migratory capacity compared to that of control cells (**Fig. 4A** and **4B**). To better analyze this phenomenon, protein levels of molecular factors associated with invasion and migration, such as E-cadherin - an epithelial marker crucial for cell-cell adhesion, as well as N-cadherin and ZEB1 - key molecules involved in cell plasticity and promotion of EMT and metastasis, were explored through western blotting. The level of E-cadherin was markedly increased in parent MCF7 cells, whereas levels of N-cadherin and ZEB1 were noticeably increased in parent TAMR cells (**Fig. 4C**). Moreover, the knockdown of HOXA5 in TAMR cells promoted a reversal of EMT by upregulating the expression of the epithelial marker, E-cadherin, and downregulating the levels of the mesenchymal markers N-cadherin and ZEB1 (**Fig. 4C**).

Increasing evidence is suggesting that a subpopulation of breast cancer stem cells contributes to the acquisition of drug resistance and ultimately metastasis and relapse in cancer patients. Hence, we evaluated whether TAMR cells display more stem-like properties when compared to MCF7 cells. We used the spheroid formation assay to analyze this phenotype. The spheroid formation assay is a method that allows the measurement of self-renewal capability and the multipotent nature of the cancer stem cell subpopulations within a cancer cell line. In the current study, the assay revealed that the size of the spheroids generated by parent TAMR cells was qualitatively more than 2-folds larger than that of parent MCF7 cells (**Fig. 4D**). To confirm that the enhanced spheroid formation was due to the elevated expression of *HOXA5* in TAMR cells, HOXA5-depleted TAMR cells were used again to assess the spheroid formation capabilities. A significant reduction of spheroids in HOXA5 knockdown TAMR cells compared to control cells was observed, supporting the role of HOXA5 involvement in cancer stemness (**Fig. 4D**). To determine whether this phenomenon is accompanied by certain underlying molecular changes, we screened the cells for differential gene expression of stem cell markers, *SOX2*, *OCT4*, and *NANOG*. All three genes were upregulated in TAMR cells, supporting the results of the spheroid formation assay. When TAMR cells were depleted of HOXA5, we observed a significant downregulation in the expression levels of *SOX2*, *OCT4*, and *NANOG* genes (**Fig. 4E**).

In summary, our data confirmed that HOXA5 plays an important role in the regulation and maintenance of aggressiveness in tamoxifen-resistant breast cancer cells by mediating invasion and migration abilities as well as stem-like characteristics.

## Discussion

In this study, we demonstrated that HOXA5 is a key molecule in activating the PI3K/AKT signaling pathway in tamoxifen-resistant breast cancer cells. We showed that it performs this role by downregulating p53 and p21 expression levels, resulting in impaired apoptosis and ultimately leading to tamoxifen resistance. We also revealed that HOXA5 contributes to breast cancer aggressiveness by modulating the expression of proteins involved in EMT. Therefore, the HOXA5/AKT/p53 axis is essential for promoting tamoxifen resistance in breast cancer.

To date, there have been contradictory observations on the role of HOXA5 in cancer. Some studies suggest that HOXA5 functions as an oncogene, whereas some suggest that it functions as a tumor suppressor 42-46. In particular, few studies have reported that HOXA5 is downregulated in breast cancer and that it functions as a tumor suppressor. Consequently, these studies showed that the overexpression of HOXA5 could prevent tumor progression and transition cancerous cells to a normal-like state 47-49. Nonetheless, the role of HOXA5 in the acquisition of tamoxifen resistance in cells has never been reported. In the current study, we suggest that targeting the overexpression of HOXA5 alone in breast cancer cells may not be therapeutically beneficial to combat tumorigenesis. In our analysis, we show evidence that high expression levels of *HOXA5* is associated with an overall poor survival in ER+ breast cancer patients who have received tamoxifen treatment (**Fig. 1C**). Therefore, based on all our observations, we propose that overexpression of HOXA5 in breast cancer may not cause the cancer cell to revert back to a normal-like state. We have also shown that the elevated expression of HOXA5 in ER+ breast cancer could result in the acquisition of tamoxifen resistance. To corroborate the clinical relevance of the elevated expression of *HOXA5* in conferring tamoxifen resistance, we used patient data retrieved from a publicly available gene expression profiling dataset (GSE1379) on 60 paired primary ER+ breast cancer patients and patients with recurrent cancer following tamoxifen monotherapy for 5 years. In agreement with the* in vitro* results, *HOXA5* expression was significantly higher in recurrent tumors, further supporting our hypothesis that *HOXA5* is a potential biomarker of tamoxifen resistance (**Fig. S3**). However, additional investigation is essential to identify in detail the precise cellular and physiological functions of *HOXA5* in the development of tamoxifen resistance.

The p53 pathway is well known to be involved in apoptosis, and its relationship with HOXA5 expression has also been reported. A previous study showed that HOXA5 functions as a direct transcriptional regulator of p53 by binding to its promoter region and also that expression levels of these two genes are positively correlated in ER+ breast cancer cells 49. In this study, however, HOXA5 and p53 expressions were negatively correlated. This apparent and contradictory observation could be explained by the fact that p53 in our tamoxifen-resistant breast cancer model system is regulated by the PI3K/AKT signaling pathway, and not directly by HOXA5 itself. This phenomenon has also been demonstrated in mice, where Hoxa5 did not alter p53 expression 50. Therefore, our results might have important implications in demonstrating that along with the expression of HOXA5, the PI3K/AKT signaling pathway needs to be simultaneously targeted to achieve efficient apoptotic effects mediated by p53.

The elevated expression of HOXA5 also induced aggressive and stem cell-like properties in TAMR cells. Currently, a growing body of evidence suggests that tamoxifen-resistant breast cancers are more invasive, metastatic, and possess the ability to self-renew and generate diversely differentiated populations in a tumor 51, 52. As a result, it is essential to study factors related to breast cancer stem cells (BCSCs) and their development. We demonstrated for the first time that HOXA5 expression is necessary for the activation of the stem cell markers (*SOX2*, *OCT4*, and *NANOG*) and the enhanced formation of spheroids enriched with BCSCs. Further examination of the mechanism and factors leading to BCSC conversion during endocrine resistance development seems crucial for HOXA5 to be applied as a therapeutic target or biomarker in patients.

**Figure 5:**
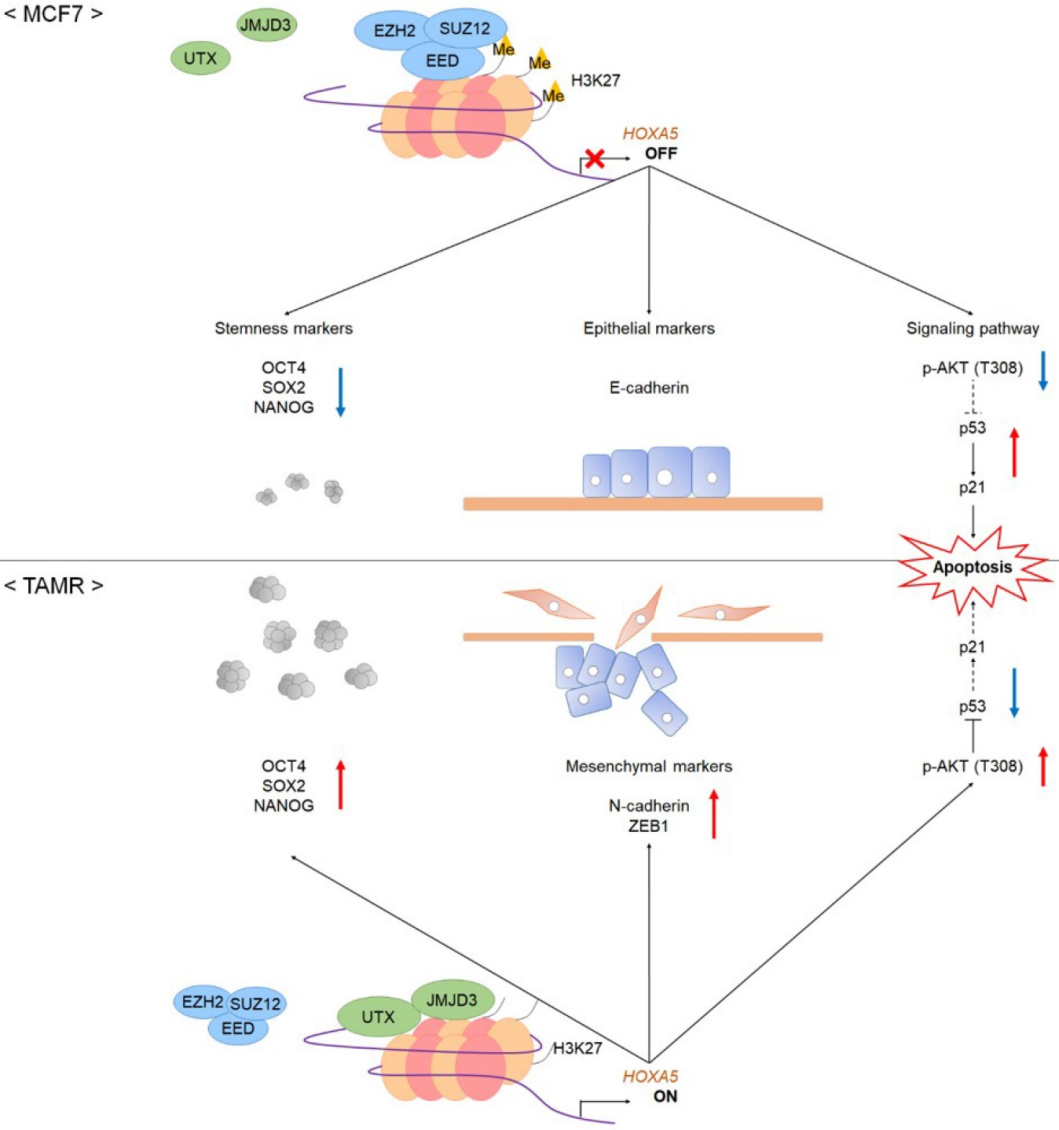
** A schematic model showing the potential role of HOXA5 in the tumorigenesis of breast cancer.** In MCF7 breast cancer cells, the PRC2 complex leads to repressive histone modifications such as H3K27me3 at the putative promoter of *HOXA5*, resulting in gene expression inhibition. This leads to apoptosis (p53 and p21 expression), epithelial phenotype (E-cadherin expression), and reduced breast cancer stem cell formation (decreased stemness marker expression). On the contrary, in TAMR cells, histone demethylases remove H3K27me3 marks at the *HOXA5* putative promoter region. This leads to diminished apoptosis (inhibition of p53 and p21 expression), mesenchymal phenotype (N-cadherin and ZEB1 expression), and enhanced breast cancer stem cell formation (increased stemness marker expression).

Our study is the first to provide evidence that HOXA5 is not only involved in tumorigenesis and/or cancer progression but is also a molecular marker for tamoxifen resistance. In this study, HOXA5 overexpression activated the PI3K/AKT signaling cascade, which in turn inhibited p53 and p21 expression, resulting in reduced apoptosis in TAMR cells. In addition, *HOXA5* induced migratory and invasive characteristics in TAMR cells by modulating the expression of epithelial and mesenchymal molecular markers. Elevated HOXA5 levels also promoted the formation of spheroids composed of BCSCs by upregulating the expression of stem cell markers (**Fig. 5**). Collectively, this study suggests that the HOXA5/AKT/p53 axis plays a crucial role in developing tamoxifen resistance and its associated phenotypes in ER+ breast cancer, and hence, could be considered as a potential therapeutic target for reversing tamoxifen resistance in breast cancer cells.

## Supplementary Material

Supplementary figures.

## Figures and Tables

**Table 1:** Primer sequences used for RT-PCR

Genes	Sequence (5′ → 3′)
*HOXA5*	F- ACC CAC ATC AGC AGC AGA GA
	R- GGC CGC CTA TGT TGT CAT
*SOX2*	F- ACA TGA ACG GCT GGA GCA
	R- GCT GCG AGT AGG ACA TGC
*OCT4*	F- CTG ATC TGC TGC AGT GTG G
	R- CCT TCC CAC CTG CAC AGA T
*NANOG*	F- CCT TCC TCC ATG GAT CTG CT
	R- TGA GGT TCA GGA TGT TGG AGA G
*β-Actin*	F- CAT GTT TGA GAC CTT CAA CAC CCC
	R- GCC ATC TCC TGC TCG AAG TCT AG

**Table 2:** Primer sequences used for ChIP-PCR assay

Amplicon sites	Sequence (5′ → 3′)
HOXA5 promoter #1	F- GCT CTC CGG AGC CAA AGT G
	R- TCA TAG TTC CGT GAG CGA GC
HOXA5 promoter #2	F- GTG CTT GAT TTG TGG CTC GC
	R- GTG ATT CGA AGT CGT ACC CCA
Gene desert	F- GAG AAG GCA CAC AGC TAG GG
	R- CCA AGC TGT ACA GGA GAG GC

## Abbreviations

HOX: homeobox
PI3K: phosphoinositide 3-kinase
AKT: protein kinase B
ER: estrogen receptor
PR: progesterone receptor
HER2: human epidermal growth factor receptor 2
SERMs: selective ER modulators
EMT: epithelial-mesenchymal transition
TAMR: tamoxifen-resistant
SOX2: sex determining region Y-box 2
OCT4: octamer-binding transcription factor 4
NANOG: nanog homeobox
BCSCs: breast cancer stem cells
ZEB1: zinc finger E-box binding homeobox 1
OS: overall survival
