# Supplementary material for: HOXA5 confers tamoxifen resistance via the PI3K/AKT signaling pathway in ER-positive breast cancer
Source: J Cancer. 2021 Jun 1;12(15):4626–37. doi: 10.7150/jca.59740 (PMC8210559; doi:10.7150/jca.59740)
Supplement: Supplementary file 1 — Supplementary figures. [file jcav12p4626s1.pdf]

Supplementary Figures

HOXA5 confers tamoxifen resistance via the PI3K/AKT signaling pathway in ER-positive breast cancer

Clara Yuri Kim[1], Yu Cheon Kim[1,2], Ji Hoon Oh[1]*, and Myoung Hee Kim[1]*

[1]*Department of Anatomy, Embryology Laboratory, Yonsei University College of Medicine, Seoul 03722, Korea*

[2]*Department of Anatomy, Graduate School of Medical Science, Bain Korea 21 Project, Yonsei University College of Medicine, Seoul 03722, Korea*

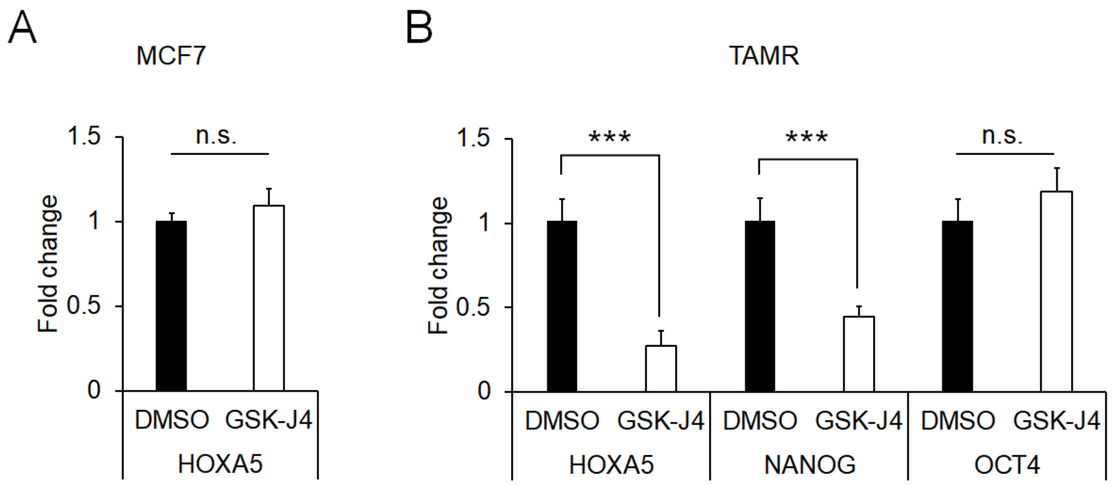

Supplementary Figure S1. JMJD3/UTX regulate the expression levels of HOXA5 in TAMR cells. (A-B) RT-qPCR analysis of HOXA5 in (A) MCF7 and (B) TAMR cells treated with GSK-J4 (JMJD3/UTX inhibitor; 5 μM). NANOG was used as a positive control and OCT4 was used as a negative control to GSK-J4 treatment. GAPDH was used to normalize changes in each gene expression level. All experiments were performed in triplicate. *** $p < 0.001$ compared with DMSO by Student's *t*-test.

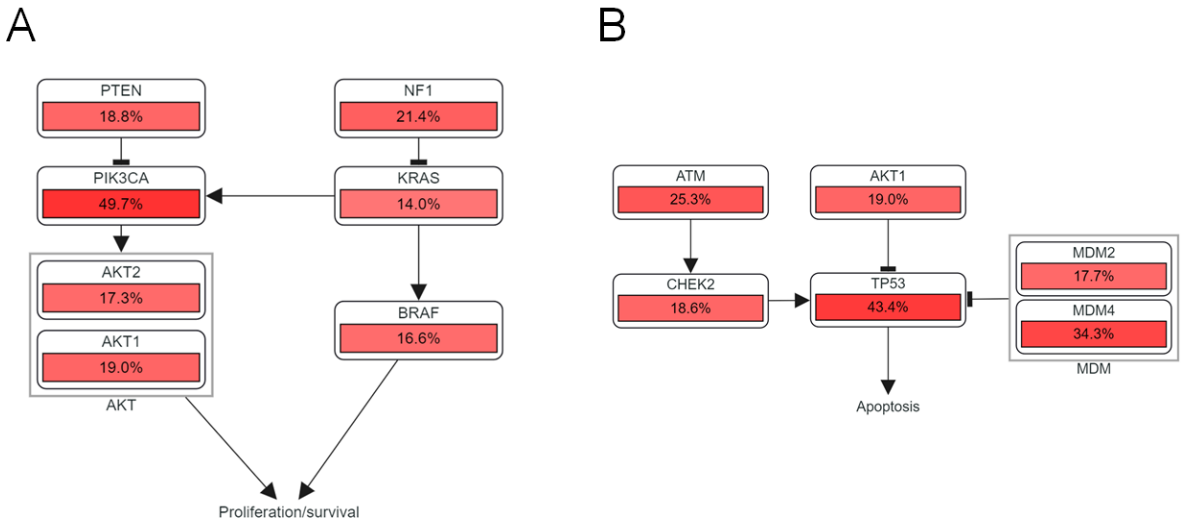

Supplementary Figure S2. Signaling pathway associated with HOXA5 alteration in breast cancer tissues. (A) PI3K/AKT signaling pathway is associated with *HOXA5* alteration in breast cancer tissues retrieved from cBioPortal. (B) TP53 pathway is associated with *HOXA5* alteration in breast cancer tissues retrieved from cBioPortal.

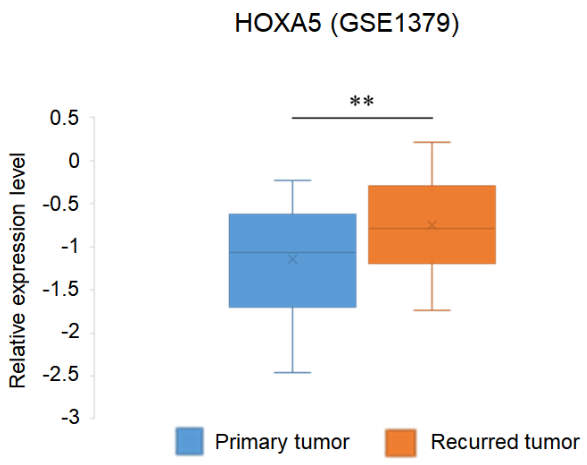

Supplementary Figure S3. Box plot of *HOXA5* expression in primary ER+ breast tumors and recurred tumors following tamoxifen mono-therapy for 5 yrs. Data were retrieved from GSE1379, a publicly available gene expression profiling data from 60 paired patients. ** $p < 0.01$ compared with primary tumor by Student's *t*-test.
